# Turning Stem Cells into Mesenchymal Tissues

**DOI:** 10.1371/journal.pmed.0020201

**Published:** 2005-06-28

As cells specialize during development they pass through different levels of differentiation, from the earliest stem cells through to the highly specialized types that make up the body's organs. Hence, a number of different tissues may derive from common precursors. For example, muscle, fat, cartilage, and bone are all derived from a group of mesenchymal precursor cells that originate in the paraxial mesoderm. So pluripotent (i.e., able to differentiate into any cell type) human embryonic stems cells are potentially a starting point for the regeneration of all types of diseased or damaged organs (and already researchers have shown that it is possible to stimulate human embryonic stem cells to differentiate into specific cell types such as neural or hematopoietic cells). The isolation of intermediate multipotent stem cells (which can differentiate into a limited number of cell types) may also be valuable. For example, the production of an unlimited supply of mesenchymal precursors would be very useful, not only for the understanding of how cells differentiate, but also for eventual practical application.

In this month's *PLoS Medicine*, Lorenz Studer and colleagues from the Sloan-Kettering Institute in New York describe a protocol for deriving mesenchymal precursors, which they then show are capable of differentiating into specialized cell types.

They used two undifferentiated stem cell lines—from the 22 lines that were approved in 2001 by President Bush for use in federally funded research in the United States. The specifications for approval for these lines are clear—see the guidelines at http://stemcells.nih.gov/research/registry/eligibilityCriteria.asp. The number of human embryonic stem cell lines available for researchers are strictly limited, making it necessary to develop protocols that expand these cells along various lineages.

In order to differentiate the cells into mesenchymal precursors, the stem cell lines were cocultured with mouse feeder cells to produce five different polyclonal lines. The authors then cultured these polyclonal precursors with appropriate tissue-specific stimulation in attempt to produce fat, bone, cartilage, or muscle cells. The evidence that the authors provide for these cells being differentiated includes analysis of gene expression, surface antigens, and immunocytochemistry typical of the mature tissues. For example, the authors were able to show the presence of fat granules in adipocytes, calcium in the matrix of osteogenic cells, and collagen in chondrocytes. It was harder to produce muscle cells, but even these types of cells could eventually be induced by specific culture conditions. [Fig pmed-0020201-g001]

**Figure pmed-0020201-g001:**
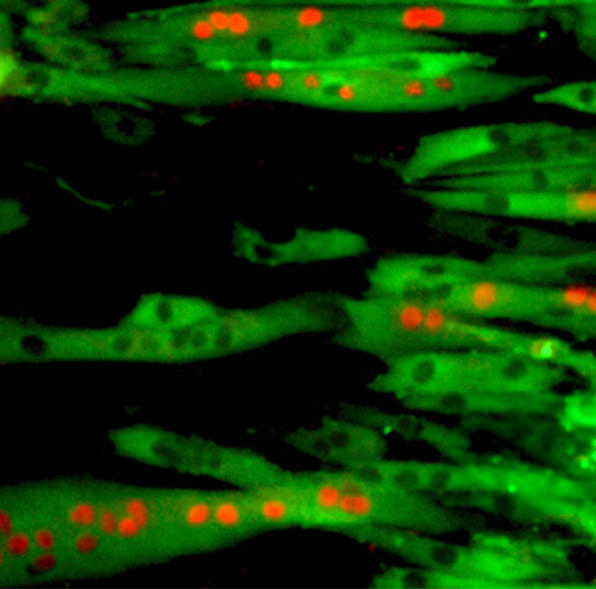
Myotubes formed in vitro from human ES–derived mesenchymal precursors upon cocultures with C2C12 myoblasts

What are the possible concerns about these types of studies? One obvious one is the potential for residual undifferentiated cells to turn into tumors, but the authors tested the differentiated cell cultures for cell surface markers characteristic of undifferentiated cells and found no evidence of them. Another worry for the use of these cells directly in humans is the need, at least at the beginning, to culture the cells with mouse feeder cells—obviously no human treatment could contain cells contaminated with mouse cells. Further development of protocols will be needed to address this issue. However, as the authors comment, “the high purity, unlimited availability, and multipotentiality of hESMPCs [human embryonic stem cell–derived mesenchymal precursor cells] will provide the basis for future therapeutic efforts using these cells in preclinical animal models of disease.” In addition, the techniques described here will provide a very useful resource for studying mesenchymal cell development.

